# Predicting Bond Strength between FRP Rebars and Concrete by Deploying Gene Expression Programming Model

**DOI:** 10.3390/polym14112145

**Published:** 2022-05-25

**Authors:** Muhammad Nasir Amin, Mudassir Iqbal, Babatunde Abiodun Salami, Arshad Jamal, Kaffayatullah Khan, Abdullah Mohammad Abu-Arab, Qasem Mohammed Sultan Al-Ahmad, Muhammad Imran

**Affiliations:** 1 Department of Civil and Environmental Engineering, College of Engineering, King Faisal University, P.O. Box 380, Al-Hofuf 31982, Al-Ahsa, Saudi Arabia; kkhan@kfu.edu.sa (K.K.); 219041496@student.kfu.edu.sa (A.M.A.-A.); 219008505@student.kfu.edu.sa (Q.M.S.A.-A.); 2 Shanghai Key Laboratory for Digital Maintenance of Buildings and Infrastructure, State Key Laboratory of Ocean Engineering, School of Naval Architecture, Ocean & Civil Engineering, Shanghai Jiao Tong University, Shanghai 200240, China; mudassiriqbal29@sjtu.edu.cn; 3 Department of Civil Engineering, University of Engineering and Technology, Peshawar 25120, Pakistan; 4 Interdisciplinary Research Center for Construction and Building Materials, Research Institute, King Fahd University of Petroleum and Minerals, P.O. Box 5040, Dhahran 31261, Saudi Arabia; salami@kfupm.edu.sa; 5 Transportation and Traffic Engineering Department, College of Engineering, Imam Abdulrahman Bin Faisal University, P.O. Box 1982, Dammam 31451, Saudi Arabia; ajjamal@iau.edu.sa; 6 School of Civil and Environmental Engineering (SCEE), National University of Sciences & Technology (NUST), Islamabad 44000, Pakistan; imran.nice@nust.edu.pk

**Keywords:** FRP, concrete compressive strength, concrete cover to bar diameter ratio, bond strength, GEP modelling, parametric study

## Abstract

Rebars made of fiber-reinforced plastic (FRP) might be the future reinforcing material, replacing mild steel rebars, which are prone to corrosion. The bond characteristics of FRP rebars differ from those of mild steel rebars due to their different stress-strain behavior than mild steel. As a result, determining the bond strength (BS) qualities of FRP rebars is critical. In this work, BS data for FRP rebars was investigated, utilizing non-linear capabilities of gene expression programming (GEP) on 273 samples. The BS of FRP and concrete was considered a function of bar surface (*Bs*), bar diameter (*d_b_*), concrete compressive strength (*f_c_*′), concrete-cover-bar-diameter ratio (*c*/*d*), and embedment-length-bar-diameter ratio (*l*/*d*). The investigation of the variable number of genetic parameters such as number of chromosomes, head size, and number of genes was undertaken such that 11 different models (M1–M11) were created. The results of accuracy evaluation parameters, namely coefficient of determination (R^2^), mean absolute error (MAE), and root mean square error (RMSE) imply that the M11 model outperforms other created models for the training and testing stages, with values of (0.925, 0.751, 1.08) and (0.9285, 0.802, 1.11), respectively. The values of R^2^ and error indices showed that there is very close agreement between the experimental and predicted results. 30 number chromosomes, 9 head size, and 5 genes yielded the optimum model. The parametric analysis revealed that *d_b_*, *c*/*d*, and *l*/*d* significantly affected the BS. The FRP rebar diameter size is greater than 10 mm, whereas a *l*/*d* ratio of more than 12 showed a considerable decrease in BS. In contrast, the rise in *c*/*d* ratio revealed second-degree increasing trend of BS.

## 1. Introduction

Numerous issues have caused the deterioration of civil engineering structures and infrastructure. Although these structures are designed to serve lives in double-digit years, many exhibit signs of distress much earlier in their service lives. Distress-induced deterioration of structures is usually caused by factors such as extreme summer and winter temperatures such as freeze and thaw cycles, and hot weather. Depending on the nature and severity of the deterioration, which might impact either the concrete or reinforcing element, or both, several remedial measures may be required. Traditional methods of rehabilitation and strengthening involve the replacement of damaged structural element repair of corroded reinforced concrete elements and the application of protective repair coatings [1]. However, there are limitations to the traditional methods. For instance, a technique that has been widely used to repair corrosion-induced concrete spalling is spot patching. This technique is limited by its inability to hinder chloride-induced corrosion, whose rates are higher around the corrosion repair sites, and its dependence on the nature of the patch material used [2]. The high cost and inefficiency of traditional repairs motivated the quest for an alternative repair solution for fiber-reinforced polymer (FRP) composite materials. Reinforcing bars, grids, sheets, and prestress tendons are just a few FRP products that are accessible to structural engineers. When new structural members are made, some of these products, such as reinforcing bars and tendons, are employed to replace steel reinforcements to improve their service lives. Steel rebar corrosion is a critical problem that compromises the performance of reinforced concrete (RC) structures. FRP rebars are among the most appealing substitutes for typical steel rebars embedded in RC structures to overcome the corrosion problem. Corrosion resistance, light weight, high strength-to-weight ratio, high-cost efficiency, ease of installation, fatigue resistance, low creep deformation, and strong chemical resistance are advantages of nonmetallic FRP rebars over steel [3,4,5]. The corrosion resistance of FRP rebars is a principal advantage, as steel-rebar corrosion has long been recognized as a significant and costly maintenance problem. Different types of FRPs have been developed and introduced in the literature [6,7,8]. Aramid, basalt, carbon, and glass fibers are normally used to make FRP rebars. Resins such as epoxy, polyester and vinyl ester are used to bind fibers together. Although FRP has greater corrosion resistance compared to the typical steel bar, the mechanical properties and long-term corrosion resistance are also highly related to the kind of FRP when facing alkaline environmental conditions of concrete penetrant [9]. Different types of FRPs shall be investigated to evaluate their resistance to corrosion and cost impact. For example, GFRP and BFRP are vulnerable to alkaline exposure due to chemical breakdown effects [10]. In comparison, CFRP exhibited outstanding mechanical behavior and corrosion resistance [11]. Varied varieties of FRPs have different costs, e.g., CFRP has a higher price than BFRP and GFRP. Moreover, FRP bars can have significantly different mechanical and physical properties, and surface profiles than those of regular steel rebars. The characteristics and the volumetric ratio of the fibers determine the elastic modulus and tensile strength of FRP bars [12]. When compared to steel rebars, the performance of such rebars is quite high when measured by their tension capacity to weight (or volume) ratio; however, their major shortcoming is poor bonding with concrete [13].

Although FRP bars have received increased attention as a reliable and feasible alternative to steel rebar against corrosion, the bond strength (BS) between concrete and FRP bars remains a subject of ongoing research [14,15]. The interfacial bond between the FRP bars and the surrounding concrete is a critical component in the performance of FRP-reinforced concrete elements under loads. Interfacial connections have been demonstrated to degrade dramatically when subjected to adverse environmental conditions in various small-scale experimental tests. A threat to its impeccable bonding property and performance is the susceptibility of its resin-rich outer layer to degradation under hygrothermal and dry heat loading [16]. Many authors [12,15,16,17] have investigated the bond between FRP rebars and concrete using a variety of bars with varying fiber qualities and quantities, as well as varying external surface designs. The FRP rebar-concrete bond is dependent on several factors, including friction attributable to the FRP rebar surface finish, mechanical adhesion of the FRP rebars against the concrete, chemical adhesion, hydrostatic pressure against the FRP rebars owing to hardened concrete shrinkage, and bulging of FRP rebars owing to temperature adjustment and moisture absorption [18,19,20,21].

One of the critical factors in reinforced concrete structural design is the BS between the reinforcing elements (FRP bars) and the reinforced (surrounding concrete). As a result, it requires accurate and precise estimation for a reliable and safe reinforced structure design. Through experimental investigations, researchers have developed empirical models for the estimation of BS of FRP while equally understanding the influence of parameters like the bonding length and compressive strength of concrete substrate on it [22,23,24,25,26]. Next, certain empirical prediction models were devised and incorporated in relevant design codes based on theoretical analysis and experimental validation. However, most of these models were developed using limited experiment datasets, which may make them exact within these data space but lack sufficient generalization capacity for other parameter settings [27,28]. An example is the standard empirical model reported in the American Concrete Institute (ACI) Committee 440 Guide for the Design and Construction of Structural Concrete Reinforced with FRP Bars that was used to traditionally estimate the BS of FRP (ACI 440.1 R-06 [29]). However, during theoretical deduction process, these constrained empirical models employed multiple assumptions to depict the complicated nonlinear relationship between BS and critical key factors, hence, reducing the model’s efficiency. It has become vital to create an accurate and computationally efficient estimation approach for FRP BS [30].

The determination of a variety of structural properties of reinforced concrete is an important issue that has piqued the interest of researchers, who have attempted to simulate them using different ML techniques [31,32,33,34]. With the advancement of computer science and the increasing volume of associated experimental datasets, data-driven approaches based on machine learning (ML) algorithms have recently emerged as alternative methods for establishing prediction models using comprehensive experimental data and information [35,36,37,38,39]. Some of the most commonly and successfully deployed ML algorithms for estimating the BS of FRP are artificial neural networks (ANNs), support vector machines (SVMs), multiple linear regression (MLR), genetic and evolutionary algorithms (GEAs), random forest (RF), and ensemble learning (gradient boosted regression trees [GBRT]) [18,27,28,35,40,41,42,43,44,45]. Thakur et al. [13] proposed a bagged M5P tree regression model out of six different models for the prediction of the bonding strength of FRP bars embedded in concrete. An ANN was also deployed in another study [45] to estimate the bonding strength of FRP bars to understand the composite behavior between the bars and concrete substrate. A new branch of genetic programming called multigene genetic programming (MGGP) was also proposed, relying on its remarkable prediction capabilities to estimate the BS of FRP bars. Considering its successful implementation and lofty performance in different studies, gene expression programming (GEP) was chosen in this study to estimate the BS of FRP [31,41,42,46,47]. Free from computational issues of slow convergence rates and local minimum convergence, GEP uses a linear constant-length expression tree (ET), a mathematical expression representation arranged in a tree-like the structure of data. GEP is a tree in which the leaves are the operands of the mathematical expression, and the nodes are the operators. GEP can tackle somewhat complicated problems with good performance by utilizing ET [41].

The objective of this study was to propose a new empirical equation to accurately predict the BS of FRP bars and concrete using a GEP-based model. For this purpose, 273 data points from previously published work were used for computational experiments. Section 2 presents the experimental data collection and description, the description of the proposed GEP-based learning model, and the experimental methodology adopted in the model training process. Section 3 reports the results of the study, comparing the predicted with the experimental results, then discusses the model performance using statistical measures in addition to parametric analysis, and, in the end, an empirical equation for the BS estimation of FRP was also developed.

## 2. Methodology

### 2.1. Experimental Database

**Figure 1:**
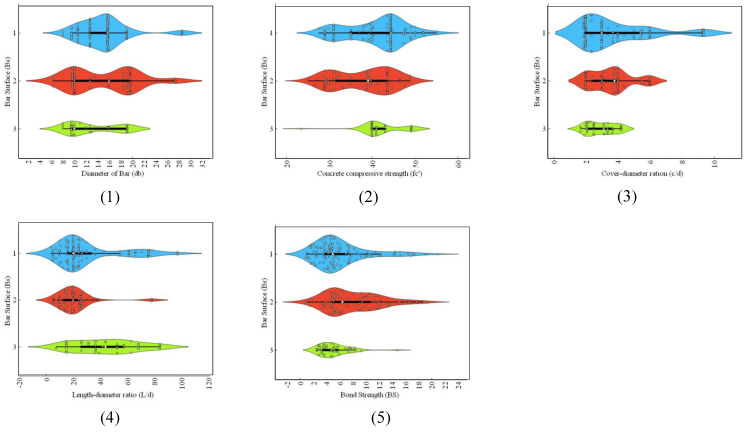
Violin frequency plots input parameters: (**1**) diameter of bar (*d_b_*), (**2**) concrete compressive strength (*f_c_*′), (**3**) concrete-cover-bar-diameter ratio (*c*/*d*), (**4**) embedment-length-bar-diameter ratio (*l*/*d*), (**5**) bond strength (BS) with respect to the bar surface (*B_s_*). Different colors shows the voilin plots distinctly for three types of bar surfaces.

**Figure 2:**
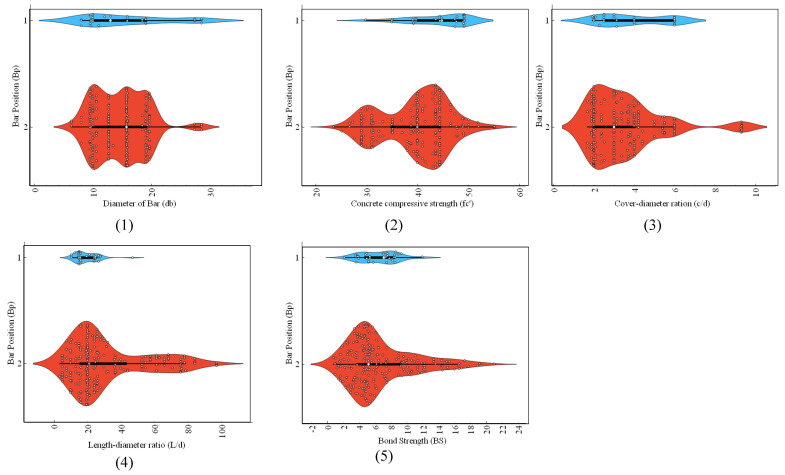
Violin frequency plots distribution of input parameters: (**1**) *d_b_*, (**2**) (*f_c_*′), (**3**) *c*/*d*, (**4**) *l*/*d*, (**5**) BS with respect to the *Bs*. Different colors shows the voilin plots distinctly for two types of bar position.

**Figure 3:**
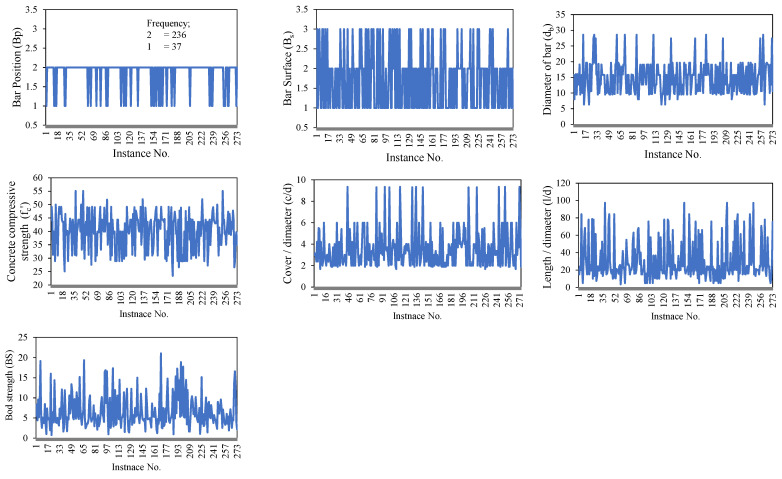
Magnitude variation of variables used in the development of models.

To build a strong ML model, it is necessary to create a short and broad database with a clear and concise description, as well as statistically evaluated input variables and information about the datasets. To this end, a comprehensive database of the required parameters for the prediction of BS of FRP was created. Details of the dataset used for the development and validation of the model, which comprises 273 experimental observations of BS of FRP concrete from published works, can be found in the study of Thakur et al. [13], also reported by Refs. [48,49,50,51]. To investigate the possible parameters governing the behavior of the BS of FRP, a thorough literature research and statistical analysis were carried out to come up with an optimized dataset for adequate evaluation. For model training and validation, the input variables were bar position (*B_p_*), bar surface (*B_s_*) condition, concrete-cover-to-bar-diameter ratio (*c*/*d*), concrete compressive strength ($f_{c}^{\prime }$), bar diameter (*d_b_*) and bar-embedment-length-to-bar-diameter ratio (*l*/*d*), and the target variable was the BS of the FRP. Table 1 lists the input and output parameters (experimental design variables) used in this study. The distribution of input and target parameters throughout model development is seen in Figure 1, Figure 2 and Figure 3. Violin plots are drawn to manifest the distribution of input variables in *B_s_*, namely helical wrapped, spiral-wrapped, and sand-coated for FRP bars (Figure 1). The box plots in each violin plot are also presented. The majority of the specimens of FRP rebars range from 10 mm to 20 mm, *f_c_*′ (30–50 MPa), *c*/*d* (2–6), and *l*/*d* within 40. A good proportion of the specimens are helically wrapped FRP rebars, whereas in most of the specimen tested, FRP rebars are located at the bottom. These graphs are especially useful since they help identify parameter values for which there is data inadequacy and additional data is needed [52].

### 2.2. Modelling Using GEP

**Figure 4:**
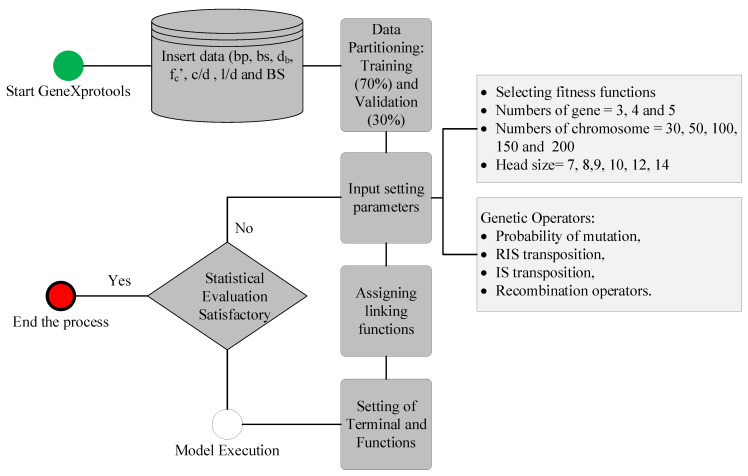
Flowchart of GEP modelling.

The GEP models were created using GeneXprotools. Initially, the data was retrieved into the interface of the tool, where the attributes were divided into target and input variables. The data was randomly partitioned into training and validation data. Previous studies showed that the partitioning in the ratios of 70/30 yielded the best performance [53,54,55,56,57,58,59,60]. Therefore, the current study adopted some partitioning percentages. In the next step, the setting parameters were changed such that number of chromosomes varied from 30 to 200, with the head size from 8 to 12, in accordance with Khan et al. [59]. The number of genes plays a vital role in the performance of the model because of the complexity of the output mathematical equation [61]. Three different numbers of genes, i.e., 3, 4, and 5, were used in the evaluation of the models in this study. A further increase in the number of genes may improve the performance; however, it may complexify the mathematical equation. The genetic operators were kept as per Iqbal et al. [39]). Different linking functions between the genes are scrutinized; however, addition yielded the best performance; therefore, it was employed in the current study. The flowchart showing GEP modelling is shown in Figure 4. The mode was executed with RMSE as the fitness function. The detail of trails is given in Table 2.

Previous studies have reported that the best parameter setting for the GEP model is based on trial and error [62,63,64,65,66]. GEP algorithm was allowed for random portioning of training and validation datasets. This way, the developed models tend to overfit during the training process and improve its performance for the training set while decreasing the performance of validation data [67]. To tackle this problem, Gandomi, A. H. and D. A. Roke [68] suggested selecting a model with a minimum objective function (OF) [69]. OF varies from 0 to the maximum, with a value approaching zero indicating a better model comparatively [49,55]. Different statistical indices such as correlation coefficient (R), root mean square error (RMSE), and mean absolute error (MAE) were used for model evaluation (Equations (1)–(3)). The R value ranges between 0 and 1, with 1 reflecting a perfect correlation, whereas values near to zero show a very weak correlation between the predictors and the target variable. The value of R equalling 0.8 and above has been generally agreed to yield a more robust and reliable prediction of the forecasted values [55,58,61,70,71,72,73,74,75].

(1) $$R=\frac{\sum_{i=1}^{n}\left(e_{i}-{\bar{e}}_{i\;}\right)\left(m_{i}-{\bar{m}}_{i\;}\right)}{\sqrt{\sum_{i=1}^{n}{\left(e_{i}-{\bar{e}}_{i\;}\right)}^{2}{\left(m_{i}-{\bar{m}}_{i\;}\right)}^{2}}},$$

(2) $$\mathrm{MAE}=\frac{\sum_{i=1}^{n}|e_{i}-m_{i}|}{n}$$

(3) $$\mathrm{RMSE}=\sqrt{\frac{\sum_{i=1}^{n}{\left(e_{i}-m_{i\;}\right)}^{2}}{n}},$$

where *e_i_* and *m_i_* are the *n*th experimental and model BS (%), respectively; $\bar{e_{i\;}}$ and $\bar{m_{i}}$ denote the average values of the experimental and model BS (%), respectively, and *n* is the number of samples in the dataset.

To find the best hyperparameters values for the current problem, a total of 11 trials (M1 to M11) were performed with varying numbers of chromosomes, head sizes, and number of genes, as shown in Table 2. Initially, chromosomes were varied from 30 to 200, keeping the head size constant at 8 and the number of genes at value of 3, which indicated that optimum model performance was achieved at a chromosome size of 8. Next, the head size was varied between 9 to 12, keeping the chromosomes (8) and genes (3) constant again. It was revealed that a head size of 9 produced the best model performance. Finally, using the above optimum values for a number of chromosomes and head size, a number of genes was varied, and the optimum model performance was obtained when number of genes was set to 5. To conclude, the proposed model yielded superior performance at parameters values of 30, 9, and 5 as the number of chromosomes, head size, and the number of genes, respectively.

## 3. Results and Discussion

This section presents the performance of models alongside the investigation of the best hyperparameters setting for the GEP model. The performance was measured in terms of statistical indices, regression slopes, and predicted to experimental ratio. Based on the accurate model, GEP formulation was achieved from the best fit model.

### 3.1. Effect of Variable Genetic Parameters

**Figure 5:**
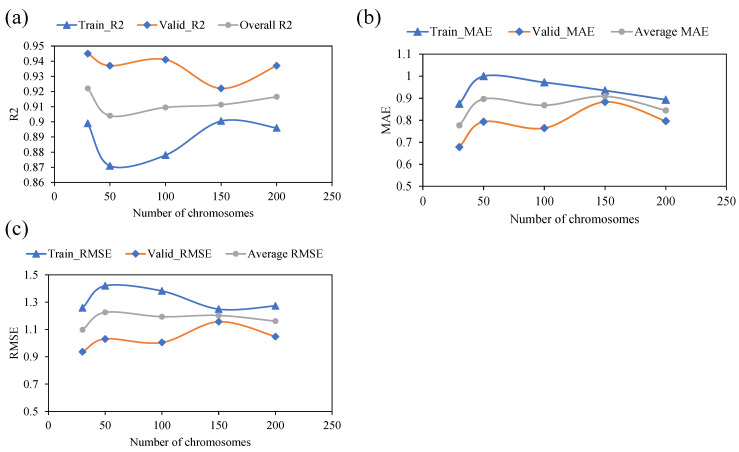
Effect of the number of chromosomes on the performance of the models: (**a**) R^2^, (**b**) MAE, and (**c**) RMSE.

Table 2 shows the model’s performance in terms of different evaluation metrics (as R^2^, RMSE, and MAE) as the number of the numbers of chromosomes are increased from 30 to 200. The trend for training, validation, and average values for the selected measures is plotted. As shown in Figure 5, the R^2^ values exhibited a downward trend as the number of chromosomes initially increased from 30 to 70. However, a further increase in the number of chromosomes leads to a corresponding considerable increase in the R^2^ correlation values. Considering the patterns of RMSE and MAE with an increasing number of chromosomes, it may be noted that both the metric showed a slight increase initially when the number of chromosomes is increased from 30 to 50. However, these metrics witnessed an overall downward trend as the number of chromosomes was further increased to 200, both for training and validation datasets. The maximum correlation and minimum error metrics were achieved at a chromosome size of 30.

**Figure 6:**
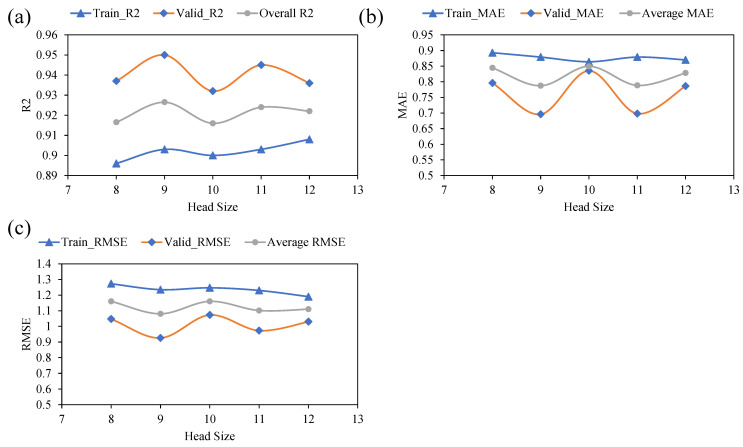
Effect of head size on the performance of the models: (**a**) R^2^, (**b**) MAE, and (**c**) RMSE.

Figure 5 depicts the model’s performance with subsequent variation in the head size this time. Again, the y-axis shows the predictive performance of the model based on the same statistical indices for both training and validation data. A similar scenario was observed with an increasing number of chromosomes and increasing head size. It may be noted from Figure 6 that an initial increase in the head size from 8 to 9 is accompanied by an increase in R values and a decrease in the values of chosen error indices. A further increase in head sizes showed a fluctuating pattern for various metrics; however, the optimum performance of the model for both training and validation data was observed at a head size of 9. Figure 6 plots the performance of the models as a function of an increase in number of genes. The results indicated that the best model performance is obtained with five numbers of genes. For the corresponding values of R^2^ for the training and validation data, it was observed that the maximum values of R^2^ were 0.92, and 0.93, respectively. Similarly, both the RSME and MAE error indices had minimum values at a gene size of five. It is worth to mention that any further increase in the number of genes may have yielded improvement in the model performance; however, this was not explored since it is likely to complexify the output mathematical relation.

**Figure 7:**
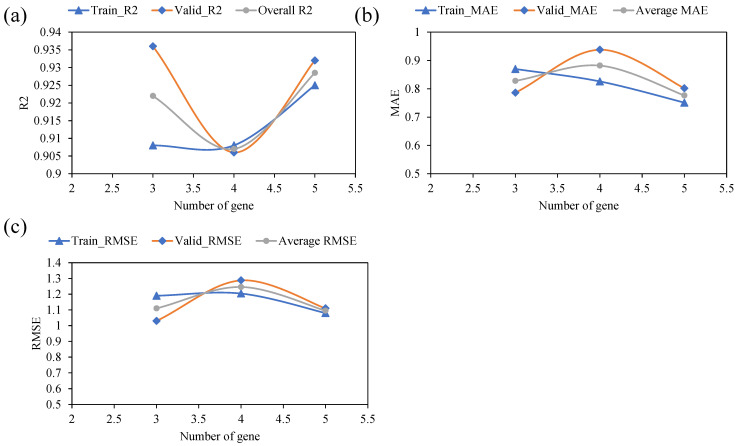
Effect of number of genes on the performance of the models: (**a**) R^2^, (**b**) MAE, and (**c**) RMSE.

In summary, it may be stated that the optimum prediction performance was obtained at chromosomes head size, and number of genes of 30, 9, and 5, respectively. Results shown in 2 and Figure 5, Figure 6 and Figure 7 for the proposed GEP model provide evidence for these observations. Recently, Mousavi et al. [76] proposed the application of the GEP model for investigating the compressive strength of high-performance concrete and reported that the model achieved the best performance at hyperparameters values of 200 as the number of chromosomes, 8 as the head size, and 3 as the optimum number of genes. It may be argued that the optimum hyperparameter setting and selection of the GEP model are dependent on trail and access method. The primary goal of hyper-parameter optimization is to achieve high R^2^ and lower values for the error indices (RMSE and MAE). Hence, this optimized model was later used for extracting the ETs and the development of mathematical equations.

### 3.2. Performance of the Models

This section is focused on the slope of the performance of the developed models in terms of the slope of the regression line, statistical evaluation, and predicted/experimental ratio (pred/exp). For the development of an efficient machine learning (ML) model, the ratio between the number of experimental records (i.e., 70% Training and 30% Validation data points, which in this case are 192 and 81, respectively) and explanatory input variable (6 number considered in the current study) must not be less than three and must preferably exceed 5 [77]. In this study, this ratio is far beyond the recommended limit (i.e., 32 in the training set and 13.5 in the validation set) for the considered BS estimation, which indicates a relatively more reliable ML model.

#### 3.2.1. Statistical Evaluation

The experimental (actual) and prediction results of the GEP model for BS of FRP bars in concrete in the training and validation stage are visualized in Table 2. The statistical evaluation shown in Table 2 manifests the value of R^2^ as significantly higher than 0.88, reflecting the close agreement of experimental to predicted results. It can be seen that M11 excels other models considering the values of R^2^. However, it is generally agreed that a higher R^2^ alone is not an exclusive and reliable indicator to assess the superiority and robustness of an artificial intelligence (AI) model [58]. Therefore, for comparison purposes, the current study considered other important indices such as RMSE and MAE, to verify the efficacy of the formulated GEP models. The GEP model prediction results based on different statistical metrics are shown in Table 2. It may be observed from the experimental results that R^2^ values for M11 models for both training and validation sets are comparable and are also greater compared to other models. The average R^2^ value for this model is the highest (0.928). A value of R^2^ greater than 0.8 shows close agreement of experimental and predicted results [78]. The corresponding values for RSME and mean MAE (0.776) are also the lowest, indicating the robustness and superior prediction performance of the M11 models. M1 is identified as the next best model. The prediction results shown in Table 2 demonstrate an acceptable performance for all the formulated GEP models. Such reliable and precise performance of the GEP model may be attributed to its algorithmic structure, which employs the diverse reproduction process for transferring appropriate data to the next stage generation and mutant operator for optimization without assuming predefined assumptions about the data [57,79]. Further, the GEP technique produces random functions and choices that agree with experimental observations [61,80,81]. In comparison to the previously developed AI models such as multilinear regression, random tree, M5P, random forest, stochastic-M5P, bagged-M5P tree, and Gaussian process, the GEP model presents comparable performance; however, it excels other AI models in terms of yielding a simple mathematical equation, whereas the previously developed models are black-box models [13].

#### 3.2.2. Comparison of Regression Slopes

**Figure 8:**
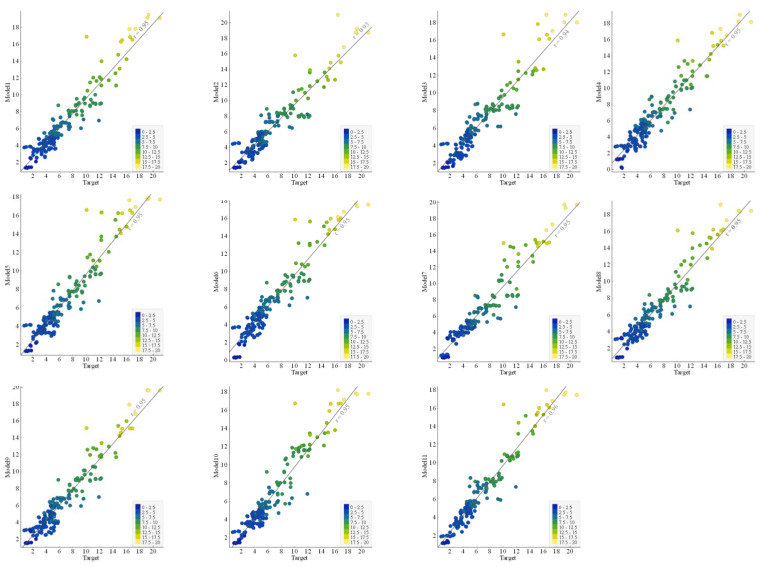
Comparison of the regression slope between experimental and predicted results for the training data.

**Figure 9:**
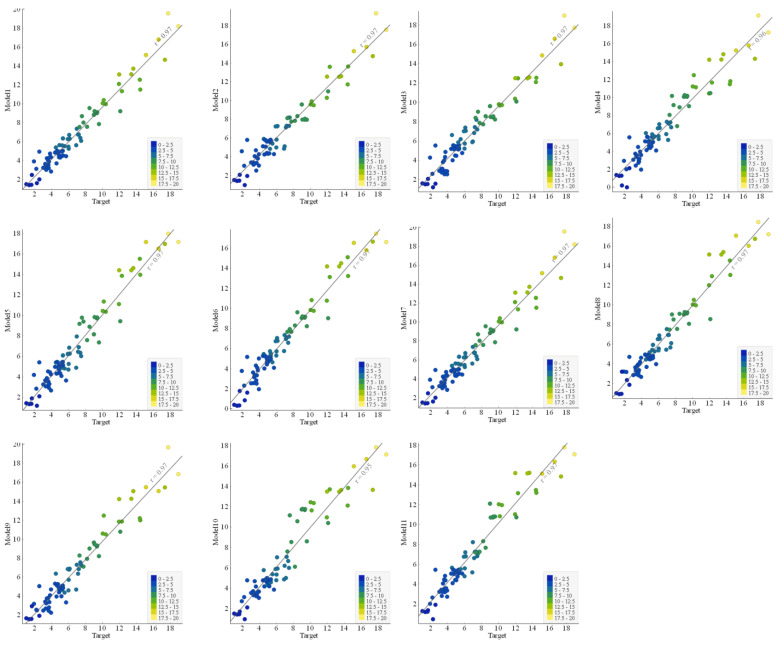
Comparison of the regression slope between experimental and predicted results for the testing data.

The regression AI models are generally evaluated using the slope of the line trending between experimental and predicted results [34,82]. This research study also reported the comparative performance of the developed 11 GEP models based on regression slopes (Figure 8 and Figure 9). The ideal fitted line having a slope equal to unity (1) is shown by standard 45 degrees passing through the diagonal. For strongly correlated lines and excellent model performance, the distribution of plotted points should be closer to the standard diagonal line. A regression line with a slope approaching 1 and correlation values of 0.8 and above will exhibit minimal values for the error indices, including RMSE and MAE [54,55,58,72,73,83]. The slope of the plotted regression line (showing the discrepancy between the target and actual BS of FRP bars) for the best model corresponds to 0.96 in the training stage and 0.97 in the validation stage. It can be seen from the plotted regression lines for different models that, in general, points are clustered around the trend line, indicating a reasonable and acceptable performance for all the models. It may be noted that both the R^2^ and regression lines slope values for the validation data are either equal or greater than those of the corresponding values for training data, showing that no overfitting issue incurred.

#### 3.2.3. Model Predicted to Experimental Ratio

**Figure 10:**
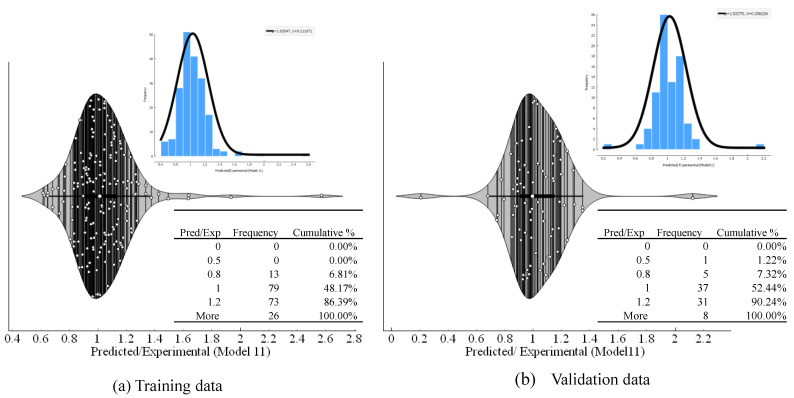
Predicted/experimental ratios of the optimum model: (**a**) Training data, and (**b**) Validation data.

The ratio of the model’s predicted results divided by the experimental results was plotted in the form of frequency ratio and cumulative Percentage (Table 3, Figure 10), specifically for the ratio between 0.8 to 1.2, which shows a 20% error in the predicted values. The maximum frequency of observations in between 0.9 and 1.1 indicates that most of the datapoints lie within ±10% error, reflecting more robust predictions. Observing the following Table 3, it can be seen that Model 11 yielded the highest cumulative of 86.39% for a bin range of 0.8 to 1.2 at training and 90.24% for the validation stage. Besides, for bin 0.8–1.0, it also gave the highest frequency equalling 79, among all the developed models. Therefore, observing the results of error indices, slopes comparison, and pred/exp comparison, the results produced for M11 are presented from this point onwards.

### 3.3. GEP Formulations

To get an empirical formulation for forecasting the BS of FRP concrete, the optimal combination of GEP parameters yielding M11 was used in accordance with the previous literature [55,58,61,83,84]. The final empirical equation is shown in Equations (4)–(9), obtained by combining the different mathematical models that were generated from the GEP model programmed in Matlab. The developed model given in the following equations is applicable for estimation of the bond strength of FRP rebars with surrounding concrete using variables; *Bs* condition, bar location, bar size, *f_c_*′, *l*/*d* ratio, and the *c*/*d* ratio. It is worth mentioning that the developed model can be used for the prediction of BS in ordinary conditions. For long-term service life in harsh environmental conditions, further studies are needed in accordance with the previous literature [85,86].

BS=A+B+C+D+E

(4) $$A\;=\sqrt[{3}]{\left(B_{p}\times \left(\left(\frac{\frac{c}{d}}{\frac{l}{d}}\right)-\left(\frac{l}{d}-3.17+B_{p}-8.39\right)\right)\right)},$$

(5) $$B=\left(\frac{\;\left(\frac{\left(d_{b}\times B_{p}\left(B_{s}-0.37\right)\right)}{\frac{c}{d}+3.50}\right)}{-0.865}\right)+6.785,$$

(6) $$C=\left(\frac{\left(-62.66-\frac{\frac{l}{d}}{\frac{c}{d}}\right)}{-2.36-\frac{l}{d}}-{f^{\prime }}_{c}\right)-\left(1.68+\sqrt[{3}]{B_{p}-8.79}\right)),$$

(7) $$D={f^{\prime }}_{c}-\frac{{f^{\prime }}_{c}\times \frac{l}{d}}{12.48\;d_{b}(d_{b}+{f^{\prime }}_{c})-d_{b}{}^{4}},$$

(8) $$E=\frac{0.566}{\left(\left(-7.17\;B_{s}-\sqrt[{3}]{-7.81)}-\left(\left(d_{b}-9.56\right)\times -4.71\right)\right)+d_{b}\right)}+B_{p},$$

where *B_p_* = position of the bar, *B_s_* = surface of the bar, *d_b_* = diameter of the rebar, *f_c_*′ = concrete compressive strength, *c*/*d* = concrete-cover-bar-diameter ratio, *l*/*d* = embedment-length-bar-diameter ratio, and BS = bond strength.

The equation can be used to predict the BS of FRPs in concrete without experiencing new experimental data, yielding the cost-effectiveness and economy of the project.

### 3.4. Parametric Analysis

To establish and verify the reliability of the ML-based simulation on diverse datasets, it is often important to check its performance on simulated datasets. Two such studies in this regard are parametric analysis and sensitivity analysis, which aim to assess the effectiveness of selected GEP models based on the interdependency of physical phenomena [58,87,88,89]. The sensitivity analysis reflects the response of the predictive model in relation to the variation of specific input features [69,88,90]. For the current study, parametric analysis was conducted to examine the respective influence of each input variable on the predicted BS of FRP concrete samples.

**Figure 11:**
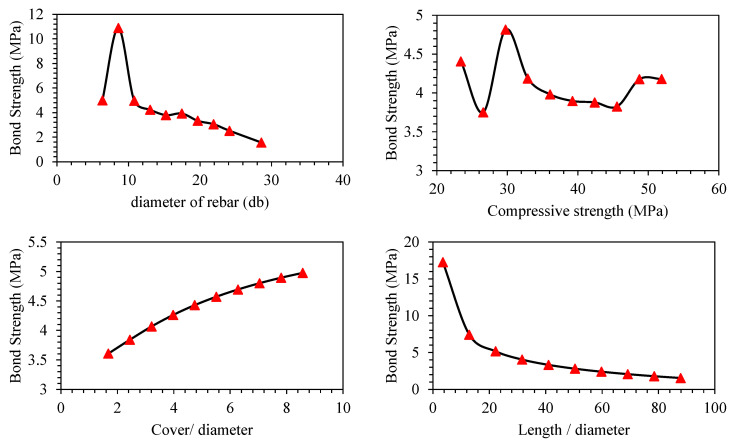
Effect of the contributing parameters on BS for bar position (*B_p_*) type-I and *B_s_* type 1.

**Figure 12:**
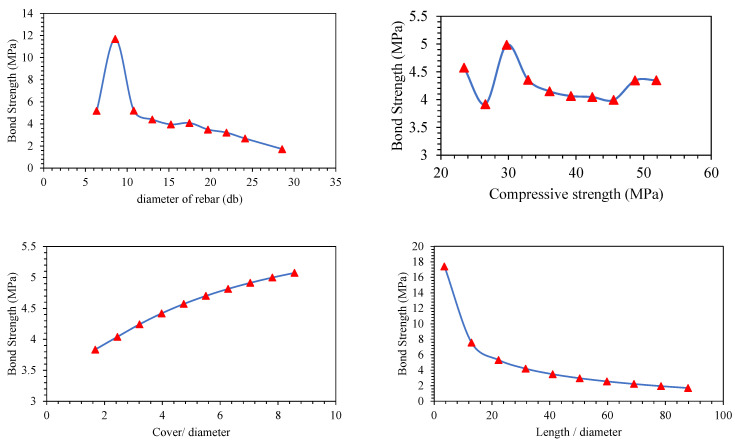
Effect of the contributing parameters on BS for *B_p_* type-I and *B_s_* type II.

**Figure 13:**
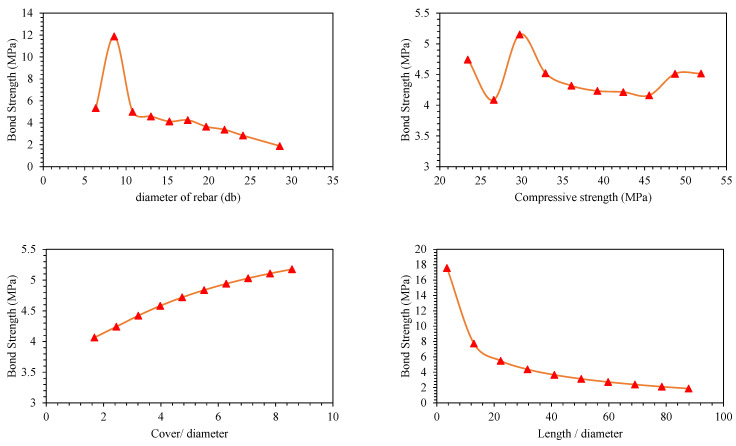
Effect of the contributing parameters on BS for *B_p_* type-I and *B_s_* type III.

**Figure 14:**
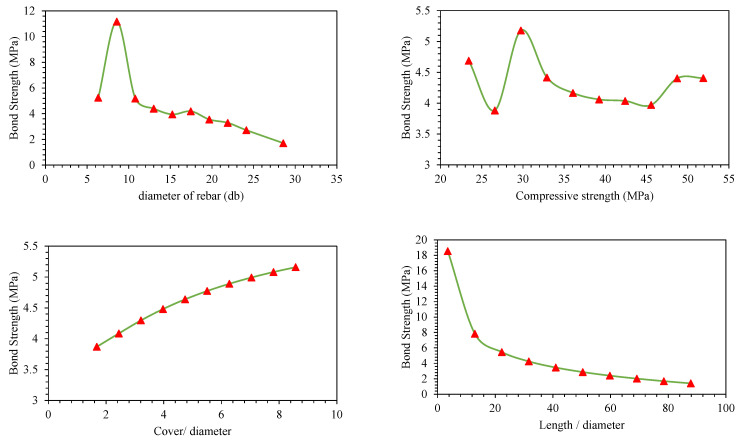
Effect of the contributing parameters on BS for *B_p_* type-II and *B_s_* type I.

**Figure 15:**
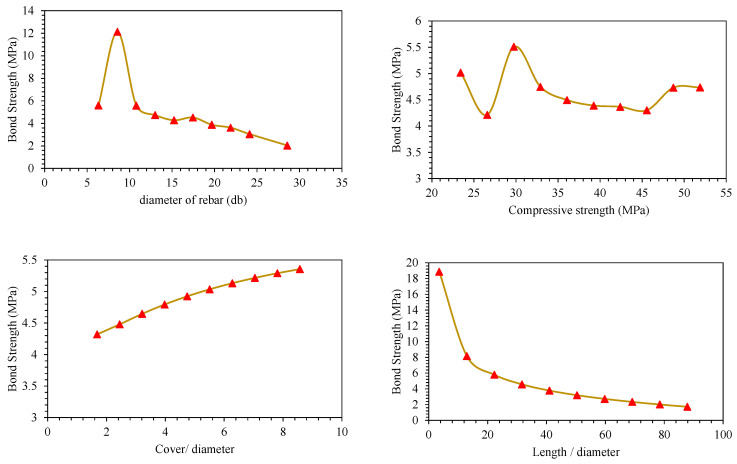
Effect of the contributing parameters on BS for *B_p_* type-II and *B_s_* type II.

**Figure 16:**
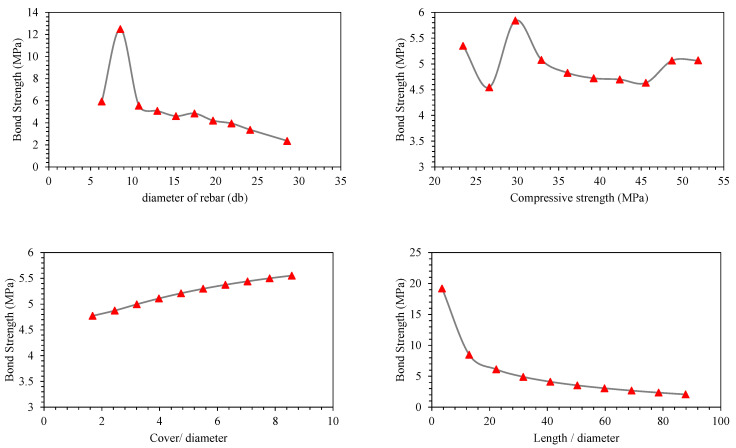
Effect of the contributing parameters on BS for *B_p_* type-II and *B_s_* type III.

Parametric analysis of all the predictors (*d_b_*, *f_c_*′, *c*/*d*, *l*/*d*) was conducted to establish their relative influence in predicting BS of FRP (graphically presented in Figure 11, Figure 12, Figure 13, Figure 14, Figure 15 and Figure 16). In addition to the above numeric input parameters, two categorical inputs, such as *B_s_* and *B_p_*, were also considered in the parametric analysis. A detailed description of three bar surfaces (type I, II, and III), and bar positions (I and II) has been provided in detail in Section 2.1. Table 4 shows the possible combination permutation of different input parameters adopted for parametric analysis. Based on *B_s_* types and bar positions, a total of six (3 × 2) groups of variable combinations are formulated. For each change, 10 data points were employed to see the effect of contributing parameters on the BS of glass FRP (GFRP) rebars in concrete. For instance, considering the first group of variable combinations (*B_s_* I and *B_p_* I) in the parametric study, initially, *d_b_* was varied between its minimum and maximum values while considering the average values of all other numeric variables. Next, the *f_c_*′ was varied between its extreme values, keeping other parameters at their mean values for this first group of bar surfaces and positions. Likewise, variables *c*/*d* and *l*/*d* were also varied to see their respective influence on the predictive performance of the GEP model. The process was repeated for other *B_s_* and *B_p_* combinations (simulated tables shown as Table 4).

Figure 11, Figure 12, Figure 13, Figure 14, Figure 15 and Figure 16 display the influence of the considered predictors, i.e., *d_b_*, *f_c_*′, *c*/*d*, and *l*/*d* for different combinations of bar surfaces and positions. As shown in Figure 11a, it is clear that the BS of FRP rebars decreases with an increase in *d_b_*. When *d_b_* is initially increased from 6 mm to 8 mm, BS is increased; however, further increase in the *d_b_* is accompanied by a steady reduction in BS. Considering the effect of *f_c_*′ on the BS of FRP rebars (Figure 11b), it may be noted that an initial increase in *f_c_*′ has some noticeable role on the target variable (BS); however, increasing *f_c_*′ beyond 35 MPa has no significant influence on the same. The variable *c*/*d* has an approximately linear correlation with the BS, i.e., any increase in *c*/*d* led to an increase in the corresponding increase in BS values. This observation is intuitive and is consistent with a number of previous studies [13,45]. Finally, considering the effect of the input predictor *l*/*d*, it may be observed that any increase in *l*/*d* is associated with a rapid decrease in BS value, and the overall pattern of strength reduction resembles an exponential curve. A number of previous studies also indicated that BS of rebars in concrete is inversely correlated with an increase in *l*/*d* [13,45]. The trends and patterns of relationships in Figure 12, Figure 13, Figure 14, Figure 15 and Figure 16 may be interpreted in the same fashion, which means that *B_s_* and *B_p_* did not change the trend considerably.

## 4. Conclusions

Due to corrosion difficulties, FRP rebars are increasingly replacing traditional steel reinforcements. The goal of this research is to develop a model for predicting the BS of FRP rebars in concrete. To calculate the BS of FRP reinforced concrete, a new prediction model in the form of a simple mathematical expression has been developed. The following findings may be taken from this investigation:

1. For the training and validation datasets, the optimum statistical indices achieved in case of the eventually selected optimal model (Trial 11) were RMSE (1.08 and 1.11), MAE (0.751 and 0.802), and R^2^ (0.932 and 0.9285), respectively. In addition, the MAE values in the constructed model show a mean error of 11.32% (training) and 12.09% (validation). These values are much lower, demonstrating the correctness and robustness of the defined GEP models for predicting BS of FRP reinforced concrete in the formulated GEP model.
2. Other statistical assessing indicators, such as (i) slope of regression line between experimental and anticipated results, (ii) predicted to experimental ratios for all models, were used to augment the GEP model performance. The best model produced regression slopes of 0.96 (training) and 0.97 (validation), which are closer to unity (i.e., ideal slope) than the others. The best trial predicted/experimental ratios revealed that 86.39% and 90.24% of the values were within 20% of each other.
3. The MATLAB code extracted from the final GEP model was used to create a mathematical equation with easily determinable input parameters to evaluate the BS of FRP reinforced concrete, avoiding the time-consuming and costly testing of samples and thus impacting the cost-effectiveness of civil engineering projects.
4. The parametric analysis revealed that a rise in *c*/*d* ratio increased the value of BS, whereas an increase in *l*/*d* decreased the BS. The *f_c_*′ was observed to have no appreciable impact on BS beyond 35 MPa. The experimental results corroborate the findings and confirm the generalization and robustness of the developed GEP models. The current GEP model may be effectively deployed for future purposes to evaluate the BS of FRP reinforced concrete.

## Figures and Tables

**Table 1:** Descriptive statistics of the database used to develop models.

Attribute Type	Input	Input	Input	Input	Output
Descriptive Statistics	Diameter of Bar (*d_b_*)	Concrete Compressive Strength (*f_c_*′)	Concrete-Cover-Bar-Diameter Ratio (*c*/*d*)	Embedment-Length-Bar-Diameter Ratio (*l*/*d*)	Bond Strength (*BS*)
Unit	mm	MPa	−	−	MPa
Mean	14.80	40.09	3.60	30.31	6.63
Standard error	0.30	0.40	0.11	1.36	0.24
Median	15.75	40.20	3.00	20.16	5.28
Mode	15.75	44.36	2.00	20.00	3.60
Standard deviation	4.98	6.61	1.82	22.43	4.01
Sample variance	24.80	43.69	3.30	503.11	16.04
Kurtosis	0.51	−0.62	2.35	0.66	1.21
Skewness	0.78	−0.37	1.53	1.31	1.24
Range	22.23	31.63	7.66	93.68	20.24
Minimum	6.35	23.43	1.68	3.56	0.76
Maximum	28.58	55.06	9.34	97.24	21.00
Sum	4039.67	10945.13	981.75	8275.05	1808.68
Count	273.00	273.00	273.00	273.00	273.00
Confidence level (95%)	0.59	0.79	0.22	2.67	0.48

**Table 2:** Details of the trials scrutinized in this study.

Model	Total Data Sets	No. of Inputs	No. of Chromosomes	Head Size	Used Variables	Number of Genes	Training Data Set			Validation Data Set				
							R^2^	RMSE	MAE	R^2^	RMSE	MAE	Overall R^2^	Overall MAE
M1	273	6	30	8	6	3	0.899	1.258	0.875	0.945	0.936	0.678	0.922	0.7765
M2	--	--	50	--	--	--	0.871	1.42	1	0.937	1.03	0.793	0.904	0.8965
M3	--	--	100	--	--	--	0.878	1.382	0.972	0.941	1.004	0.764	0.9095	0.868
M4	--	--	150	--	--	--	0.9005	1.249	0.935	0.922	1.156	0.883	0.91125	0.909
M5	--	--	200	--	--	--	0.896	1.273	0.893	0.937	1.047	0.796	0.9165	0.8445
M6	--	--	30	9	--	--	0.903	1.235	0.879	0.95	0.926	0.696	0.9265	0.7875
M7	--	--	--	10	--	--	0.9	1.247	0.864	0.932	1.073	0.835	0.916	0.8495
M8	--	--	--	11	--	--	0.903	1.23	0.879	0.945	0.973	0.698	0.924	0.7885
M9	--	--	--	12	--	--	0.908	1.19	0.87	0.936	1.03	0.786	0.922	0.828
M10	--	--	--	9	--	4	0.908	1.204	0.826	0.906	1.288	0.938	0.907	0.882
M11	--	--	--	9	--	5	0.925	1.08	0.751	0.932	1.11	0.802	0.9285	0.7765

Note: (--) shows the same value of the setting parameter as the one in the above cell.

**Table 3:** Comparison of frequency ratios of predicted to experimental values for the developed models.

**Model 1**			**Model 2**			**Model 3**			**Model 4**		
Pred/Exp	Frequency	Cumulative %	Pred/Exp	Frequency	Cumulative %	Pred/Exp	Frequency	Cumulative %	Pred/Exp	Frequency	Cumulative %
0	0	0.00%	0	0	0.00%	0	0	0.00%	0	0	0.00%
0.5	0	0.00%	0.5	0	0.00%	0.5	0	0.00%	0.5	2	1.05%
0.8	16	8.38%	0.8	27	14.14%	0.8	31	16.23%	0.8	25	14.14%
1	68	43.98%	1	58	44.50%	1	59	47.12%	1	64	47.64%
1.2	76	83.77%	1.2	75	83.77%	1.2	62	79.58%	1.2	65	81.68%
More	31	100.00%	More	31	100.00%	More	39	100.00%	More	35	100.00%
**Model 5**			**Model 6**			**Model 7**			**Model 8**		
Pred/Exp	Frequency	Cumulative %	Pred/Exp	Frequency	Cumulative %	Pred/Exp	Frequency	Cumulative %	Pred/Exp	Frequency	Cumulative %
0	0	0.00%	0	0	0.00%	0	0	0.00%	0	0	0.00%
0.5	0	0.00%	0.5	5	2.62%	0.5	0	0.00%	0.5	0	0.00%
0.8	16	8.38%	0.8	18	12.04%	0.8	15	7.85%	0.8	24	12.57%
1	70	45.03%	1	65	46.07%	1	72	45.55%	1	73	50.79%
1.2	76	84.82%	1.2	72	83.77%	1.2	72	83.25%	1.2	67	85.86%
More	29	100.00%	More	31	100.00%	More	32	100.00%	More	27	100.00%
**Model 9**			**Model 10**			**Model 11**					
Pred/Exp	Frequency	Cumulative %	Pred/Exp	Frequency	Cumulative %	Pred/Exp	Frequency	Cumulative %			
0	0	0.00%	0	0	0.00%	0	0	0.00%			
0.5	0	0.00%	0.5	0	0.00%	0.5	0	0.00%			
0.8	22	11.52%	0.8	18	9.42%	0.8	13	6.81%			
1	65	45.55%	1	67	44.50%	1	79	48.17%			
1.2	69	81.68%	1.2	78	85.34%	1.2	73	86.39%			
More	35	100.00%	More	28	100.00%	More	26	100.00%			

**Table 4:** Simulated dataset for parametric analysis.

Variable Input Parameters		No. of Datapoints	Constant Input Parameters
Parameter	Range		
*d_b_*	6.35–28.58	10	*B_p_* = I, B_s_ = I, *f_c_*′= 40.09, *c*/*d* = 3.59, *l*/*d* = 30.31
*f_c_*′	23.43–55.06	10	*B_p_* = I, B_s_ = I, *d_b_*_=_ 14.79, *c*/*d* = 3.59, *l*/*d* = 30.31
*c*/*d*	1.68–9.34	10	*B_p_* = I, B_s_ = I, *d_b_*_=_ 14.79, *f_c_*′ = 40.09, *l*/*d* = 30.31
*l*/*d*	3.56–97.25	10	*B_p_* = I, B_s_ = I, *d_b_*_=_ 14.79, *f_c_*′ = 40.09, *c*/*d* = 3.59
*d_b_*	6.35–28.58	10	*B_p_* = I, B_s_ = II, *f_c_*′= 40.09, *c*/*d* = 3.59, *l*/*d* = 30.31
*f_c_*′	23.43–55.06	10	*B_p_* = I, B_s_ = II, *d_b_*_=_ 14.79, *c*/*d* = 3.59, *l*/*d* = 30.31
*c*/*d*	1.68–9.34	10	*B_p_* = I, B_s_ = II, *d_b_*_=_ 14.79, *f_c_*′ = 40.09, *l*/*d* = 30.31
*l*/*d*	3.56–97.25	10	*B_p_* = I, B_s_ = II, *d_b_*_=_ 14.79, *f_c_*′ = 40.09, *c*/*d* = 3.59
*d_b_*	6.35–28.58	10	*B_p_* = I, B_s_ = III, *f_c_*′= 40.09, *c*/*d* = 3.59, *l*/*d* = 30.31
*f_c_*′	23.43–55.06	10	*B_p_* = I, B_s_ = III, *d_b_*_=_ 14.79, *c*/*d* = 3.59, *l*/*d* = 30.31
*c*/*d*	1.68–9.34	10	*B_p_* = I, B_s_ = III, *d_b_*_=_ 14.79, *f_c_*′ = 40.09, *l*/*d* = 30.31
*l*/*d*	3.56–97.25	10	*B_p_* = I, B_s_ = III, *d_b_*_=_ 14.79, *f_c_*′ = 40.09, *c*/*d* = 3.59
*d_b_*	6.35–28.58	10	*B_p_* = II, B_s_ = I, *f_c_*′= 40.09, *c*/*d* = 3.59, *l*/*d* = 30.31
*f_c_*′	23.43–55.06	10	*B_p_* = II, B_s_ = I, *d_b_*_=_ 14.79, *c*/*d* = 3.59, *l*/*d* = 30.31
*c*/*d*	1.68–9.34	10	*B_p_* = II, B_s_ = I, *d_b_*_=_ 14.79, *f_c_*′ = 40.09, *l*/*d* = 30.31
*l*/*d*	3.56–97.25	10	*B_p_* = II, B_s_ = I, *d_b_*_=_ 14.79, *f_c_*′ = 40.09, *c*/*d* = 3.59
*d_b_*	6.35–28.58	10	*B_p_* = II, B_s_ = II, *f_c_*′= 40.09, *c*/*d* = 3.59, *l*/*d* = 30.31
*f_c_*′	23.43–55.06	10	*B_p_* = II, B_s_ = II, *d_b_*_=_ 14.79, *c*/*d* = 3.59, *l*/*d* = 30.31
*c*/*d*	1.68–9.34	10	*B_p_* = II, B_s_ = II, *d_b_*_=_ 14.79, *f_c_*′ = 40.09, *l*/*d* = 30.31
*l*/*d*	3.56–97.25	10	*B_p_* = II, B_s_ = II, *d_b_*_=_ 14.79, *f_c_*′ = 40.09, *c*/*d* = 3.59
*d_b_*	6.35–28.58	10	*B_p_* = II, B_s_ = III, *f_c_*′= 40.09, *c*/*d* = 3.59, *l*/*d* = 30.31
*f_c_*′	23.43–55.06	10	*B_p_* = II, B_s_ = III, *d_b_*_=_ 14.79, *c*/*d* = 3.59, *l*/*d* = 30.31
*c*/*d*	1.68–9.34	10	*B_p_* = II, B_s_ = III, *d_b_*_=_ 14.79, *f_c_*′ = 40.09, *l*/*d* = 30.31
*l*/*d*	3.56–97.25	10	*B_p_* = II, B_s_ = III, *d_b_*_=_ 14.79, *f_c_*′ = 40.09, *c*/*d* = 3.59

## Data Availability

The data used for the development of models has been reported in the paper.
